# An Extended Kalman Filter with Remainder Terms and Correlation Compensation for Nonlinear State Monitoring and Soft Sensing

**DOI:** 10.3390/s26092736

**Published:** 2026-04-28

**Authors:** Jinhao Ke, Chenglin Wen

**Affiliations:** School of Automation, Guangdong University of Petrochemical Technology, Maoming 525000, China; 13232161102@gdupt.edu.cn

**Keywords:** extended Kalman filter, nonlinear systems, soft sensing, state monitoring, truncation errors, remainder terms

## Abstract

In networked sensing systems, nonlinear state monitoring and soft sensing are widely used to reconstruct key variables that cannot be directly measured in real time. For such nonlinear estimation tasks, the Extended Kalman Filter (EKF) is a commonly used recursive method. However, the conventional EKF neglects higher-order truncation terms during first-order Taylor linearization. As the nonlinearity increases, these neglected terms may accumulate and degrade filtering accuracy, and even lead to divergence in some cases. In addition, the statistical influence of the remainder terms and the correlation between prediction and measurement errors are usually ignored. To address these issues, this paper proposes an Extended Kalman Filter with remainder terms considering correlations (REKF). The proposed method replaces the higher-order terms in the Taylor expansion with remainder terms and identifies them incrementally by using least squares, thereby improving the EKF update process. A higher-order filtering framework is then constructed to jointly estimate the system state and the remainder-related random variables while accounting for the induced error correlation. Numerical simulations on typical nonlinear models demonstrate that the proposed REKF achieves better estimation performance than the conventional EKF. In this work, the proposed REKF is mainly developed for nonlinear estimation problems in which the dominant challenge arises from strong nonlinearity in the state evolution, while the measurement update is treated in a locally linearized EKF form. The results show that incorporating higher-order remainder information can effectively improve nonlinear state estimation for state monitoring and soft sensing tasks.

## 1. Introduction

State estimation plays an important role in modern monitoring and control systems. In many networked sensing environments, such as distributed industrial monitoring platforms and intelligent process supervision systems, some key internal variables cannot be directly measured in real time because of sensor cost, installation constraints, communication limitations, or the lack of suitable physical sensing mechanisms. Therefore, these variables must be reconstructed online from accessible measurements, which is commonly referred to as nonlinear state monitoring or soft sensing [1]. Since soft sensing performance directly affects monitoring reliability, fault warning capability, and control quality, accurate recursive estimation of hidden states has become a fundamental problem in networked sensing systems [2,3].

Early Wiener filtering (WF) [4], developed in the frequency domain, was mainly used for stationary random processes. However, most real-world processes are non-stationary. To address this issue, the Kalman Filter (KF) provides optimal linear minimum mean square error estimation for linear Gaussian systems [5] and has been widely applied in signal processing and sensor fusion [6,7]. However, many practical sensing systems exhibit nonlinear dynamics [8]. The Extended Kalman Filter (EKF) [9] addresses this issue by linearizing nonlinear functions via first-order Taylor expansion, enabling recursive real-time implementation on embedded platforms [10,11,12,13,14,15].

Despite its effectiveness, the standard first-order EKF neglects higher-order terms in the Taylor expansion [16,17]. Under strong nonlinear motion, high dynamic conditions, or noisy measurements, truncation errors accumulate and may cause biased estimation, degraded accuracy, or even divergence [18,19,20]. Furthermore, the statistical characteristics of these remainder terms are usually ignored, and the correlation between prediction and measurement errors induced by nonlinear approximation is not explicitly modeled.

To enhance nonlinear approximation capability, filters such as the Unscented Kalman Filter (UKF) [21,22] and the Cubature Kalman Filter (CKF) [23] improve second-order accuracy through deterministic sampling [24,25,26,27,28]. Nevertheless, incorporating higher-order truncation effects into recursive filtering remains challenging, particularly in high-precision sensing applications. Meanwhile, Particle Filtering (PF) is applicable to systems with strong nonlinearity and non-Gaussian characteristics. However, due to the particle propagation and resampling processes, it usually incurs a significantly higher computational burden, which may limit its application in real-time scenarios. Recursive Gaussian filtering is still superior in real-time implementation, but the approximate accuracy of the first-order extended Kalman filter is insufficient. By enhancing the handling of nonlinear effects while maintaining the recursive structure, Extended Kalman Filter with Remainder Terms Considering Correlations (REKF) achieves a better balance between estimation accuracy and computational efficiency. It should be noted that higher-order information can also be incorporated by STT-based HEKF methods through explicit higher-order derivatives or tensor expansions [29,30]. In contrast, the proposed REKF achieves higher-order compensation by incremental remainder-term identification and correlation modeling, without explicit tensor construction [31,32].

To address these limitations, this paper proposes an Extended Kalman Filter with Remainder Terms Considering Correlations (REKF). Instead of discarding higher-order Taylor terms, the proposed method replaces them with remainder terms identified incrementally using a least squares approach. By incorporating the statistical properties of these remainder terms and explicitly modeling the induced correlation between prediction and measurement errors, the REKF enhances nonlinear approximation capability while preserving recursive structure.

The main contribution of this work lies in the development of a higher-order Extended Kalman Filter framework that explicitly addresses truncation effects and error correlations neglected in conventional EKF. Specifically, a remainder-term identification mechanism is established to capture higher-order Taylor truncation errors [33], and a correlation-aware filtering structure is introduced to model the coupling between prediction and measurement errors induced by nonlinear approximation. By integrating these components within the recursive Kalman filtering framework, the proposed REKF enhances nonlinear approximation accuracy while preserving real-time implementation capability, thereby significantly improving estimation robustness and precision in nonlinear sensor systems.

The main innovations of this work can be summarized as follows: (1) A remainder-term augmentation mechanism is introduced to compensate for the higher-order truncation neglected by first-order EKF linearization. (2) A correlation-aware covariance update scheme is developed to explicitly account for the coupling between prediction and measurement errors induced by remainder-term estimation. (3) Through simulations under progressively increasing nonlinearity, the proposed REKF demonstrates clear advantages, particularly in regimes where the first-order EKF exhibits noticeable performance degradation.

## 2. EKF and EKF with Remainder Terms Considering Correlations (REKF)

### 2.1. Basic Principle of the EKF

The Extended Kalman Filter (EKF) performs state estimation through recursive calculations. At each time step, the state estimate depends on the previous estimate and the current observation data. This recursive approach allows the filter to continuously and real time update the system’s state. Specifically, each calculation only relies on the most recent observation data and the previous state estimate [34], which effectively updates the state. When linearizing the system, EKF employs a first-order Taylor expansion to approximate the nonlinear system, but it neglects second-order as well as higher-order terms in the process. This simplification converts the originally nonlinear system into a linear model, simplifying the computation. However, this also leads to some loss of accuracy, as the neglected higher-order terms may contain more precise details about the system dynamics [35].

In typical EKF applications, the state equation is nonlinear, while the measurement equation is often represented in a linear or locally linearized form for recursive estimation. In this paper, although the general model in Definition 1 allows a nonlinear measurement equation, the subsequent derivation emphasizes the truncation effects caused by strong nonlinearity in the state equation. By applying a local linearization method, EKF can handle nonlinear state equations, thereby reducing estimation errors caused by system nonlinearity to some extent. However, neglecting higher-order terms in the Taylor expansion may introduce linearization errors, which result from the omission of those terms and can affect the final estimation accuracy. Therefore, this paper specifically highlights the impact of truncation errors on filter precision and suggests methods for optimizing the filter algorithm by analyzing these errors, thereby reducing estimation errors caused by linear approximation and further improving filter accuracy.

**Definition** **1.** *Consider the following stochastic differential equation in a stationary form.*

(1) $$\begin{array}{c} x_{n+1}=f\left(x_{n}\right)+\omega_{n} \end{array}$$

(2) $$\begin{array}{c} y_{n+1}=h\left(x_{n+1}\right)+\upsilon_{n+1} \end{array}$$

Let f(·) be the state equation (SE) as well as h(·) the measurement equation (ME). The time range is denoted by *T*, $x_{n+1}$ represents the n-dimensional state vector, and $y_{n+1}$ is the m-dimensional measurement vector. The system errors are modeled as two independent Gaussian noise vectors: $\omega_{n}$ for process noise as well as $\upsilon_{n+1}$ for measurement noise. The state equation $f(x_{n})$ is a nonlinear function (NF) describing the evolution of $x_{n}$ under the influence of both $\omega_{n}$ as well as $\upsilon_{n+1}$, with continuous derivatives up to the r-th order. The measurement equation $h(x_{n+1})$ is similarly nonlinear and reflects the interaction of $x_{n+1}$ as well as $\upsilon_{n+1}$. Both $\omega_{n}$ as well as $\upsilon_{n+1}$ are independent and Gaussian-distributed with zero mean. Their covariance matrices, $Q_{n}$ and $R_{n+1}$, are the statistical properties of the process as well as measurement noise. The matrix $Q_{n}$ describes the process noise, while R captures the uncertainty in measurements. These noise models provide the necessary input for filtering algorithms to account for random disturbances in system operation. They meet the following statistical characteristics:

$$E\{\omega_{n}\}=0,E\left\{\omega_{n}{\omega_{n}}^{T}\right\}=Q_{n};$$

$$E\left\{\upsilon_{n+1}\right\}=0,E\left\{\upsilon_{n+1}\upsilon_{n+1}^{T}\right\}=R_{n+1};$$

$$E\left\{\omega_{n}\upsilon_{n+1}^{T}\right\}=0,E\left\{\upsilon_{n+1}\omega_{n}^{T}\right\}=0;$$

$$E\{\omega_{n}{x_{n}}^{T}\}=0,E\{\upsilon_{n+1}{x_{n}}^{T}\}=0;$$

The filtered estimate at time *n*, ${\hat{x}}_{n|n}$ denoted as, is obtained by performing a Taylor expansion of the nonlinear function $f(x_{n})$ at ${\hat{x}}_{n|n}$ as shown in Equation (3).

(3) $$\begin{array}{c} \begin{array}{c} x_{n+1}=f\left({\hat{x}}_{n|n}\right)+\sum_{l=1}^{r}{\left.\frac{1}{l!}\frac{\partial^{l}f\left({\hat{x}}_{n|n}\right)}{\partial {x_{n}}^{l}}\right|}_{x_{n}={\hat{x}}_{n|n}}{\left[x_{n}-{\hat{x}}_{n|n}\right]}^{l}\\ \;\;\;\;\;\;\;\;+{\left.\frac{1}{(r+1)!}\frac{\partial^{r+1}f\left({\hat{x}}_{n|n}\right)}{\partial {x_{n}}^{r+1}}\right|}_{x_{n}=\xi_{n}}{\left[x_{n}-{\hat{x}}_{n}\right]}^{r+1}+\omega_{n} \end{array} \end{array}$$

Neglecting second-order and higher-order terms:

(4) $$\begin{array}{c} f\left(x_{n}\right)\approx f\left({\hat{x}}_{n|n}\right)+{\left.\frac{\partial f({\hat{x}}_{n|n})}{\partial x_{n}}\right|}_{x_{n}={\hat{x}}_{n|n}}\left[x_{n}-{\hat{x}}_{n|n}\right] \end{array}$$

x(k+1) can be expressed approximately as:

(5) $$\begin{array}{c} \begin{array}{c} x_{n+1}\approx f\left({\hat{x}}_{n|n}\right)+A_{n+1|n}\left[x_{n}-{\hat{x}}_{n|n}\right]+\omega_{n}\\ \;\;\;\;\;\;\;\approx \Delta f\left({\hat{x}}_{n|n}\right)+A_{n+1|n}x_{n}+\omega_{n} \end{array} \end{array}$$

In Equation (5):

(6) $$\begin{array}{c} A_{n+1|n}={\left.\frac{\partial f({\hat{x}}_{n|n})}{\partial x_{n}}\right|}_{x_{n}={\hat{x}}_{n|n}} \end{array}$$

where

(7) $$\begin{array}{c} \Delta f\left({\hat{x}}_{n|n}\right)=f\left({\hat{x}}_{n|n}\right)-A_{n+1|n}{\hat{x}}_{n|n} \end{array}$$

Extending the prediction and update process of the KF:

From Equation (1), the predicted estimate as well as the prediction error of the state equation in the model can be attained:

Predicted estimate:

(8) $$\begin{array}{c} {\hat{x}}_{n+1|n}\approx A_{n+1|n}{\hat{x}}_{n|n}+\Delta f\left({\hat{x}}_{n|n}\right) \end{array}$$

It follows that

(9) $$\begin{array}{c} {\tilde{x}}_{n+1|n}\approx A_{n+1|n}{\tilde{x}}_{n|n}+\omega_{n} \end{array}$$

Prediction error:

(10) $$\begin{array}{c} x_{n+1}\approx \Delta f\left({\hat{x}}_{n|n}\right)+A_{n+1|n}x_{n}+\omega_{n} \end{array}$$

From Equation (2), the measurement estimate (ME) as well as the measurement estimation error (MEE) of the measurement equation in the model can be attained:

Measurement estimate:

(11) $$\begin{array}{c} {\hat{y}}_{n+1|n}\approx H_{n+1}{\hat{x}}_{n+1|n} \end{array}$$

Measurement estimation error:

(12) $$\begin{array}{c} {\tilde{y}}_{n+1|n}\approx H_{n+1}{\tilde{x}}_{n+1|n}+\upsilon_{n+1} \end{array}$$

Prediction error covariance:

(13) $$\begin{array}{c} P_{n+1|n}\approx A_{n+1|n}P_{n|n}{A_{n+1|n}}^{T}+Q_{n} \end{array}$$

The filtered values of the Kalman observer with time-varying gain are as follows:

(14) $$\begin{array}{c} x_{n+1|n+1}\approx {\hat{x}}_{n+1|n}+K_{n+1}\left({\tilde{y}}_{n+1|n}\right) \end{array}$$

In accordance with the orthogonality principle $E\{{\tilde{x}}_{n+1|n+1}{y^{T}}_{n+1}\}=0$, the Kalman gain $K_{n+1}$ is solved as:

(15) $$\begin{array}{c} K_{n+1}\approx P_{n+1|n}\left(xx\right){H_{n+1}}^{T}{\left(H_{n+1}P_{n+1|n}\left(xx\right){H_{n+1}}^{T}+R_{n+1}\right)}^{-1} \end{array}$$

The estimation error covariance (EEC) of the state variable $x_{n+1}$ is calculated as:

(16) $$\begin{array}{c} P_{n+1|n+1}\left(xx\right)\approx \left(I-K_{n+1}H_{n+1}\right)P_{n+1|n}\left(xx\right) \end{array}$$

In the standard EKF, the statistical properties of the initial state $x_{0}$ are known: $E\left\{x_{0}\right\}={\hat{x}}_{0}$, $E\left\{\left(x_{0}-{\hat{x}}_{0}\right){\left(x_{0}-{\hat{x}}_{0}\right)}^{T}\right\}=P_{0}$.

### 2.2. EKF with Remainder Terms (REKF)

Built upon the Taylor expansion of the EKF, further consideration is given to the higher-order terms in the state equation’s Taylor expansion. By using the remainder terms to replace the higher-order terms of the Taylor expansion, the model’s accuracy can be improved. The remainder terms, are progressively identified via the least squares. REKF, built upon the original EKF, incorporates more parameters that are typically discarded in EKF, leading to a higher accuracy compared to EKF. Accordingly, the remainder-term identification and correlation compensation developed below are primarily intended to improve the prediction and filtering performance when the dominant source of approximation error comes from the nonlinear state dynamics.

#### 2.2.1. Replace the Higher-Order Terms of the Taylor Expansion of the Nonlinear State Equation with the Remainder Term

**Assumption** **1.** *The equation of state $x_{n+1}$ in Equation (1) is continuous up to the order derivative of r, as well as the order derivative of r+1 is present*.

Assumption 1 holds, and the Taylor expansion of the nonlinear function $f(x_{n})$ at ${\hat{x}}_{n|n}$ is given by:

(17) $$\begin{array}{c} \begin{array}{c} x_{n+1}=f\left({\hat{x}}_{n|n}\right)+\sum_{l=1}^{r}{\left.\frac{1}{l!}\frac{\partial^{l}f\left({\hat{x}}_{n|n}\right)}{\partial {x_{n}}^{l}}\right|}_{x_{n}={\hat{x}}_{n|n}}{\left[x_{n}-{\hat{x}}_{n|n}\right]}^{l}\\ \;\;\;\;\;\;\;\;+{\left.\frac{1}{(r+1)!}\frac{\partial^{r+1}f\left({\hat{x}}_{n|n}\right)}{\partial {x_{n}}^{r+1}}\right|}_{x_{n}=\xi_{n}}{\left[x_{n}-{\hat{x}}_{n}\right]}^{r+1}+\omega_{n} \end{array} \end{array}$$

In the state equation, ξ(k) is leveraged to substitute the second-order as well as higher-order terms:

(18) $$\begin{array}{c} \begin{array}{c} x_{n+1}=f\left(x_{n}\right)+\omega_{n}\\ \;\;\;\;\;\;\;\;=f\left({\hat{x}}_{n|n}\right)+A_{n+1|n}\left(x_{n}-{\hat{x}}_{n|n}\right)\\ \;\;\;\;\;\;\;\;+\xi_{n}+\omega_{n} \end{array} \end{array}$$

The remainder terms in the state equation are identified using the least squares method, and the identification process is as follows:

Predicted estimate and prediction error of the nonlinear function $f(x_{n})$ expanded at ${\hat{x}}_{n|n}$ using the Taylor series:

(19) $${\hat{x}}_{n+1|n}=f\left({\hat{x}}_{n|n}\right)-A_{n+1|n}{\hat{x}}_{n|n}+A_{n+1|n}{\hat{x}}_{n|n}+{\hat{\xi }}_{n|n}$$

(20) $$\begin{array}{c} {\tilde{x}}_{n+1|n}=A_{n+1|n}{\tilde{x}}_{n|n}+{\tilde{\xi }}_{n|n}+\omega_{n} \end{array}$$

Substitute Equation (2) into Equation (18):

(21) $$\begin{array}{c} \begin{array}{c} y_{n+1}=H_{n+1}f\left({\hat{x}}_{n|n}\right)+H_{n+1}A_{n}{\tilde{x}}_{n|n}\\ \;\;\;\;\;\;\;+H_{n+1}\xi_{n}+H_{n+1}\omega_{n}+\upsilon_{n+1} \end{array} \end{array}$$

Obtained through item transfer:

$$y_{n+1}-H_{n+1}f\left({\hat{x}}_{n|n}\right)=H_{n+1}\xi_{n}+H_{n+1}A_{n}{\tilde{x}}_{n|n}+H_{n+1}\omega_{n}+\upsilon_{n+1}$$

Simplify Equation (21) to obtain Equation (22):

(22) $$\begin{array}{c} {\bar{y}}_{n+1}=H_{n+1}\xi_{n}+{\bar{\upsilon }}_{n+1} \end{array}$$

where:

$${\bar{y}}_{n+1}=y_{n+1}-H_{n+1}f\left({\hat{x}}_{n|n}\right)$$

$${\bar{\upsilon }}_{n+1}=H_{n+1}A_{n}{\tilde{x}}_{n|n}+H_{n+1}\omega_{n}+\upsilon_{n+1}$$

${\bar{\upsilon }}_{n+1}$ follows a Gaussian distribution with mean 0 and variance $\bar{R}\left(k+1\right)$:

$$\begin{array}{c} \bar{R}=E\left\{\bar{\upsilon }{\bar{\upsilon }}^{T}\right\}\\ \;\;\;=H_{n+1}A_{n}P_{n}{A^{T}}_{n}{H^{T}}_{n+1}+H_{n+1}Q{H^{T}}_{n+1}\\ \;\;\;+R+H_{n+1}S_{wv}+S_{vw}{H^{T}}_{n+1} \end{array}$$

where

$$P_{n}=E\left\{{\tilde{x}}_{n|n}{\tilde{x}}_{n|n}^{T}\right\}$$

$$S_{vw}=E\left\{\upsilon_{n+1}{\omega^{T}}_{n}\right\}=0$$

$$S_{wv}=E\left\{\omega_{n}{\upsilon^{T}}_{n+1}\right\}=0$$

Note: $\omega_{n}$∼$N(0,Q_{n})$ and $\upsilon_{n+1}$∼$N(0,R_{n+1})$ are independent and follow a Gaussian distribution.

Identify ξ(k) in Equation (22) via the least squares approach

$$\xi_{n}={\hat{\xi }}_{n|n}+{\tilde{\xi }}_{n|n}$$

$${\hat{\xi }}_{n}={H^{T}}_{n+1}{\left[H_{n+1}{H^{T}}_{n+1}\right]}^{-1}{\bar{y}}_{n+1}$$

Note: The least-squares solution in Equation (22) requires that the matrix $H_{n+1}{H^{T}}_{n+1}$ be invertible, which implies that $H_{n+1}$ is of full row rank. This condition corresponds to a sufficient excitation requirement.

The convergence of the least squares estimation depends on the continuous excitation bar, that is, the observation matrix $H_{n+1}$ is required to satisfy:

$$\sum_{k=n}^{n+N}H_{k+1}^{T}H_{k+1}≻\alpha I\left(\alpha >0,N<\infty \right)$$

(23) $$\begin{array}{c} \begin{array}{c} {\tilde{\xi }}_{n}=-{H^{T}}_{n+1}{\left[H_{n+1}{H^{T}}_{n+1}\right]}^{-1}\bar{\upsilon }\\ \;\;\;\;=M_{n+1}\left(\tilde{x},1\right){\tilde{x}}_{n|n}+M_{n+1}\left(\omega ,2\right)\omega_{n}\\ \;\;\;\;+M_{n+1}\left(\upsilon ,3\right)\upsilon_{n+1} \end{array} \end{array}$$

where

$$M_{n+1}\left(\tilde{x},1\right)=-{H^{T}}_{n+1}{\left[H_{n+1}{H^{T}}_{n+1}\right]}^{-1}H_{n+1}A_{n}$$

$$M_{n+1}\left(\omega ,2\right)=-{H^{T}}_{n+1}{\left[H_{n+1}{H^{T}}_{n+1}\right]}^{-1}H_{n+1}$$

$$M_{n+1}\left(\upsilon ,3\right)=-{H^{T}}_{n+1}{\left[H_{n+1}{H^{T}}_{n+1}\right]}^{-1}$$

Note: In Equation (23), ${\tilde{\xi }}_{n}$ is formed by a linear combination of ${\tilde{x}}_{n|n}$,$\omega_{n}$, as well as $\upsilon_{n+1}$.

#### 2.2.2. Extended Kalman Filter with Remainder Terms Considering Correlations Performs Prediction and Update

The nonlinear observation as well as state equation have been linearized; an EKF with Remainder Terms Considering Correlations (REKF) can be designed to perform the prediction and update steps:

The state and observation equations of a class of nonlinear function system models are as follows:

(24) $$\begin{array}{c} x_{n+1}=f\left(x_{n}\right)+\omega_{n}\\ \;\;\;\;\;\;\;=\Delta f\left({\hat{x}}_{n}\right)+A_{n+1|n}x_{n}+\xi_{n}+\omega_{n} \end{array}$$

(25) $$y_{n+1}=H_{n+1}x_{n+1}+\upsilon_{n+1}$$

Prediction step of REKF:

Step 1: Predicted estimate of the state equation:

(26) $${\hat{x}}_{n+1|n}=\Delta f\left({\hat{x}}_{n|n}\right)+A_{n+1|n}{\hat{x}}_{n|n}+{\hat{\xi }}_{n|n}$$

Prediction error of the state equation:

(27) $$\begin{array}{c} {\tilde{x}}_{n+1|n}=x_{n+1}-{\hat{x}}_{n+1|n}\\ \;\;\;\;\;\;\;\;\;=A_{n}{\tilde{x}}_{n}+{\tilde{\xi }}_{n}+\omega_{n}\\ \;\;\;\;\;\;\;\;\;=A_{n}{\tilde{x}}_{n}+M_{n+1}\left(\tilde{x},1\right){\tilde{x}}_{n|n}+M_{n+1}\left(\omega ,2\right)\omega_{n}\\ \;\;\;\;\;\;\;\;\;+M_{n+1}\left(\upsilon ,3\right)\upsilon_{n+1}+\omega_{n}\\ \;\;\;\;\;\;\;\;\;=\left(A_{n}+M_{n+1}\left(\tilde{x},1\right)\right){\tilde{x}}_{n|n}+\left(M_{n+1}\left(\omega ,2\right)+I\right)\omega_{n}\\ \;\;\;\;\;\;\;\;\;+M_{n+1}\left(v,3\right)\upsilon_{n+1}\\ \;\;\;\;\;\;\;\;\;={\bar{M}}_{n+1}\left(\tilde{x},1\right){\tilde{x}}_{n|n}+{\bar{M}}_{n+1}\left(\omega ,2\right)\omega_{n}\\ \;\;\;\;\;\;\;\;\;+{\bar{M}}_{n+1}\left(\upsilon ,3\right)\upsilon_{n+1} \end{array}$$

Here, ${\tilde{\xi }}_{n}$ represents the estimated error of the remainder terms in the state equation, and ${\hat{\xi }}_{n}$ represents the estimated values of the remainder terms in the state equation.

Where

$$\begin{array}{c} {\bar{M}}_{n+1}\left(\tilde{x},1\right)=A_{n}+M_{n+1}\left(\tilde{x},1\right)\\ \;\;\;\;\;\;\;\;\;\;\;\;\;\;\;\;\;=A_{n}-{H^{T}}_{n+1}{\left[H_{n+1}{H^{T}}_{n+1}\right]}^{-1}H_{n+1}A_{n}\\ {\bar{M}}_{n+1}\left(w,2\right)=M_{n+1}\left(\omega ,2\right)+I\\ \;\;\;\;\;\;\;\;\;\;\;\;\;\;\;\;\;\;=-{H^{T}}_{n+1}{\left[H_{n+1}{H^{T}}_{n+1}\right]}^{-1}H_{n+1}+I\\ {\bar{M}}_{n+1}(v,3)=M_{n+1}\left(v,3\right)\\ \;\;\;\;\;\;\;\;\;\;\;\;\;\;\;\;\;=-{H^{T}}_{n+1}{\left[H_{n+1}{H^{T}}_{n+1}\right]}^{-1} \end{array}$$

Among them, ${\tilde{x}}_{n|n}$, $v_{n+1}$, and $w_{n}$ are independent of each other:

$$\begin{array}{c} E\left\{{\tilde{x}}_{n|n}w_{n}^{T}\right\}=0,E\left\{w_{n}{\tilde{x}}_{n|n}^{T}\right\}=0;\\ E\left\{{\tilde{x}}_{n|n}v_{n+1}^{T}\right\}=0,E\left\{v_{n+1}{\tilde{x}}_{n|n}^{T}\right\}=0;\\ E\left\{w_{n}v_{n+1}^{T}\right\}=0,E\left\{v_{n+1}w_{n}^{T}\right\}=0; \end{array}$$

Step 2: Prediction error covariance $P_{n+1|n}$

(28) $$\begin{array}{c} P_{n+1|n}=E\left\{{\tilde{x}}_{n+1|n}{{\tilde{x}}^{T}}_{n+1|n}\right\}\\ \;\;\;\;\;\;\;\;\;\;\;={\bar{M}}_{n+1}\left(\tilde{x},1\right)P_{n|n}{{\bar{M}}_{n+1}}^{T}\left(\tilde{x},1\right)+{\bar{M}}_{n+1}\left(\omega ,2\right)Q_{n}{{\bar{M}}_{n+1}}^{T}\left(\omega ,2\right)\\ \;\;\;\;\;\;\;\;\;\;\;+M_{n+1}\left(\upsilon ,3\right)R_{n+1}{M_{n+1}}^{T}\left(\upsilon ,3\right) \end{array}$$

Here, $P_{n|n}=E\left\{{\tilde{x}}_{n|n}{{\tilde{x}}_{n|n}}^{T}\right\}$ represents the estimation error covariance at time *k*.

From Equation (26), the predicted estimate of the measurement estimate is:

(29) $${\hat{y}}_{n+1|n}=H_{n+1}{\hat{x}}_{n+1|n}$$

Prediction error of the measurement estimate:

(30) $$\begin{array}{c} {\tilde{y}}_{n+1|n}=y_{n+1}-{\hat{y}}_{n+1|n}\\ \;\;\;\;\;\;\;\;\;=H_{n+1}{\tilde{x}}_{n+1}+\upsilon_{n+1}\\ \;\;\;\;\;\;\;\;\;=H_{n+1}{\bar{M}}_{n+1}\left(\tilde{x},1\right){\tilde{x}}_{n|n}+H_{n+1}{\bar{M}}_{n+1}\left(\omega ,2\right)\omega_{n}\\ \;\;\;\;\;\;\;\;\;+\left[H_{n+1}{\bar{M}}_{n+1}\left(\upsilon ,3\right)+I\right]\upsilon_{n+1}\\ \;\;\;\;\;\;\;\;\;={\bar{N}}_{n+1}\left(\tilde{x},1\right){\tilde{x}}_{n|n}+{\bar{N}}_{n+1}\left(\omega ,2\right)\omega_{n}+{\bar{N}}_{n+1}\left(\upsilon ,3\right)\upsilon_{n+1} \end{array}$$

where

$${\bar{N}}_{n+1}\left(\tilde{x},1\right)=H_{n+1}{\bar{M}}_{n+1}\left(\tilde{x},1\right)$$

$${\bar{N}}_{n+1}\left(\omega ,2\right)=H_{n+1}{\bar{M}}_{n+1}\left(\omega ,2\right)$$

$${\bar{N}}_{n+1}\left(\upsilon ,3\right)=H_{n+1}{\bar{M}}_{n+1}\left(\upsilon ,3\right)+I$$

Update step of REKF

Autocovariance matrix:

(31) $$\begin{array}{c} P_{n+1|n}\left(\tilde{y}\tilde{y}\right)=E\left\{{\tilde{y}}_{n+1|n}{{\tilde{y}}^{T}}_{n+1|n}\right\}\\ \;\;\;\;\;\;\;\;\;\;\;\;\;\;\;\;\;=E\{\left[{\bar{N}}_{n+1}\left(\tilde{x},1\right){\tilde{x}}_{n|n}+{\bar{N}}_{n+1}\left(\omega ,2\right)\omega_{n}+{\bar{N}}_{n+1}\left(\upsilon ,3\right)\upsilon_{n+1}\right)]\\ \;\;\;\;\;\;\;\;\;\;\;\;\;\;\;\;\;{\left[{\bar{N}}_{n+1}\left(\tilde{x},1\right){\tilde{x}}_{n|n}+{\bar{N}}_{n+1}\left(\omega ,2\right)\omega_{n}+{\bar{N}}_{n+1}\left(\upsilon ,3\right)\upsilon_{n+1}\right]}^{T}\}\\ \;\;\;\;\;\;\;\;\;\;\;\;\;\;\;\;\;={\bar{N}}_{n+1}P_{n+1|n}\left(xx\right){\bar{N}}_{n+1}^{T}+{\bar{N}}_{n+1}\left(\omega ,2\right)Q{\bar{N}}_{n+1}^{T}\left(\omega ,2\right)H_{n+1}\\ \;\;\;\;\;\;\;\;\;\;\;\;\;\;\;\;\;+{\bar{N}}_{n+1}\left(\upsilon ,3\right)R{\bar{N}}_{n+1}^{T}\left(\upsilon ,3\right){\bar{M}}_{n+1}\left(\upsilon ,3\right) \end{array}$$

Cross-covariance matrix:

(32) $$\begin{array}{c} P_{n+1|n}\left(\tilde{x}\tilde{y}\right)=E\left\{{\tilde{x}}_{n+1|n}{{\tilde{y}}^{T}}_{n+1|n}\right\}\\ \;\;\;\;\;\;\;\;\;\;\;\;\;\;\;\;\;=E\{\left[{\bar{M}}_{n+1}\left(\tilde{x},1\right){\tilde{x}}_{n|n}+{\bar{M}}_{n+1}\left(\omega ,2\right)\omega_{n}+{\bar{M}}_{n+1}\left(\upsilon ,3\right)\upsilon_{n+1}\right]\\ \;\;\;\;\;\;\;\;\;\;\;\;\;\;\;\;\;{\left[{\bar{N}}_{n+1}\left(\tilde{x},1\right){\tilde{x}}_{n|n}+{\bar{N}}_{n+1}\left(\omega ,2\right)\omega_{n}+{\bar{N}}_{n+1}\left(\upsilon ,3\right)\upsilon_{n+1}\right]}^{T}\}\\ \;\;\;\;\;\;\;\;\;\;\;\;\;\;\;\;\;\;={\bar{M}}_{n+1}\left(\tilde{x},1\right)P_{n+1|n}{\bar{N}}_{n+1}^{T}\left(\tilde{x},1\right)+{\bar{M}}_{n+1}\left(\omega ,2\right)Q{\bar{N}}_{n+1}^{T}\left(\omega ,2\right)\\ \;\;\;\;\;\;\;\;\;\;\;\;\;\;\;\;\;\;+{\bar{M}}_{n+1}\left(\upsilon ,3\right)R{\bar{N}}_{n+1}^{T}\left(\upsilon ,3\right) \end{array}$$

(33) $$P_{n+1|n}\left(\tilde{y}\tilde{x}\right)={P_{n+1|n}}^{T}\left(\tilde{x}\tilde{y}\right)$$

Step 3: Updates the Kalman gain $K_{n+1}$

(34) $$K_{n+1}=P_{n+1|n}\left(\tilde{x}\tilde{y}\right)P_{n+1|n}{\left(\tilde{y}\tilde{y}\right)}^{-1}$$

Step 4: State estimate

(35) $${\hat{x}}_{n+1}={\hat{x}}_{n+1|n}+K_{n+1}{\tilde{y}}_{n+1|n}$$

Step 5: Updated error covariance matrix:

(36) $$P_{n+1|n+1}=P_{n+1|n}-K_{n+1}P_{n+1|n}\left(yx\right)$$

**Figure 1:**
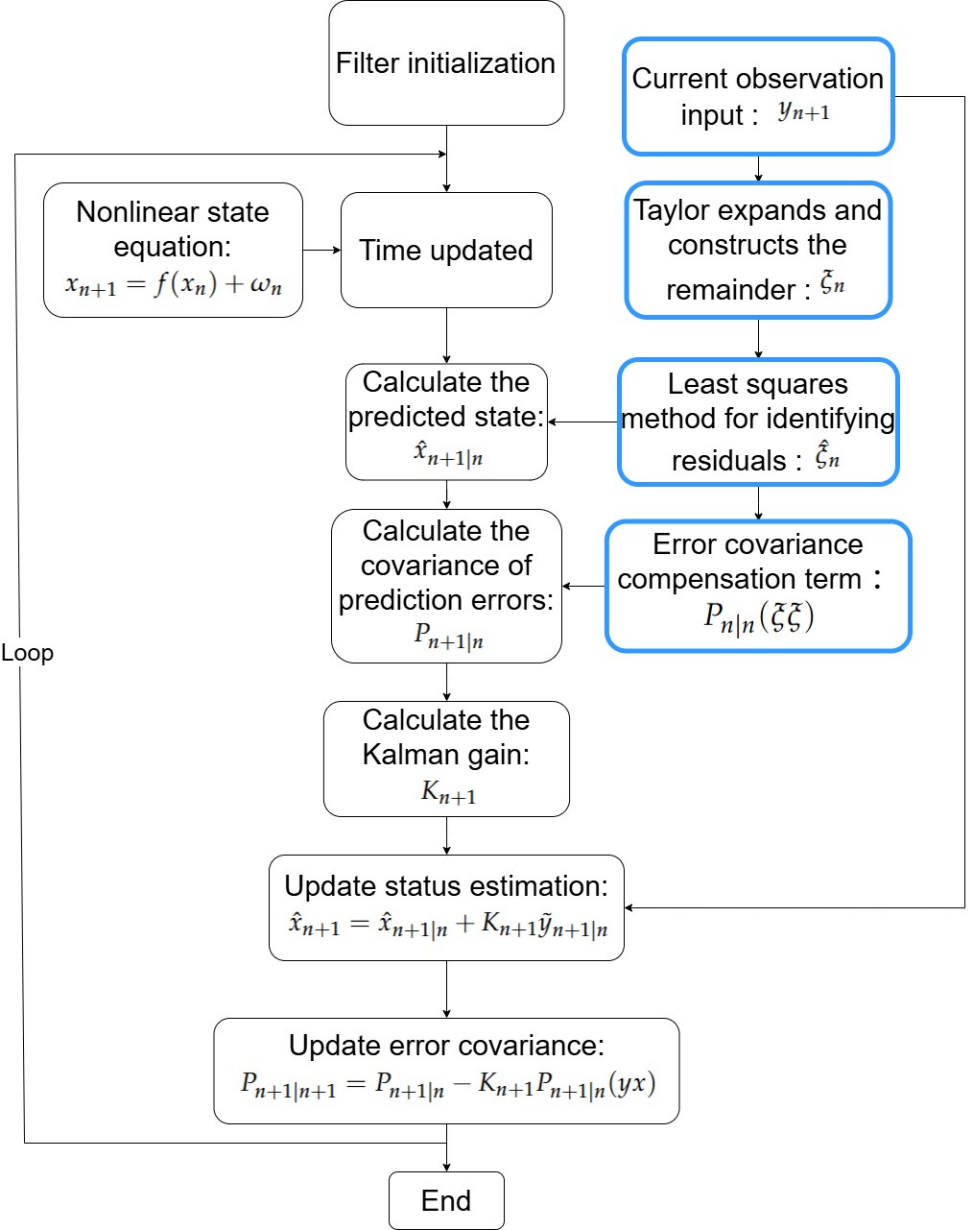
Algorithm flowchart of the sequential algorithm.

Figure 1 illustrates the flowchart of the Extended Kalman Filter with Remainder Term:

## 3. Performance Analysis of the Prediction Phase

Predicted values as well as error values during the Taylor expansion of EKF:

$$x_{n+1}=\Delta f\left({\hat{x}}_{n|n}\right)+A_{n+1}x_{n}+\xi_{n}+\omega_{n}$$

$${\hat{x}}_{n+1|n}=\Delta f\left({\hat{x}}_{n|n}\right)+A_{n+1|n}{\hat{x}}_{n|n}$$

$$\begin{array}{c} \begin{array}{c} {\tilde{x}}_{n+1|n}=x_{n+1}-{\hat{x}}_{n+1|n}\\ \;\;\;\;\;\;\;\;\;=A_{n+1|n}{\tilde{x}}_{n|n}+{\tilde{\xi }}_{n|n}+{\hat{\xi }}_{n|n}+w_{n} \end{array} \end{array}$$

Error covariance during the Taylor expansion of EKF:

$$\begin{array}{c} P_{n+1|n}\left(EKF\right)=E\left\{{\tilde{x}}_{n+1|n}{{\tilde{x}}^{T}}_{n+1|n}\right\}\\ \;\;\;\;\;\;\;\;\;\;\;\;\;\;\;\;\;\;\;\;\;\;\;=A_{n+1|n}P_{n|n}A_{n+1|n}^{T}+P_{\left(n|n\right)}\left(\xi \xi \right)+{\hat{\xi }}_{\left(n|n\right)}{{\hat{\xi }}^{T}}_{\left(n|n\right)}+Q_{\left(n\right)} \end{array}$$

Predicted values and error values during the Taylor expansion of REKF:

$$\begin{array}{c} x_{\left(n+1\right)}=\Delta f\left({\hat{x}}_{\left(n|n\right)}\right)+A_{\left(n+1|n\right)}x_{\left(n\right)}+\xi_{\left(n\right)}+w_{\left(n\right)} \end{array}$$

$$\begin{array}{c} {\hat{x}}_{\left(n+1|n\right)}=\Delta f\left({\hat{x}}_{\left(n|n\right)}\right)+A_{\left(n+1|n\right)}{\hat{x}}_{\left(n|n\right)}+{\hat{\xi }}_{\left(n|n\right)} \end{array}$$

$$\begin{array}{c} {\tilde{x}}_{\left(n+1|n\right)}=x_{\left(n+1\right)}-{\hat{x}}_{\left(n+1|n\right)}\\ \;\;\;\;\;\;\;\;\;\;\;=A_{\left(n+1|n\right)}{\tilde{x}}_{\left(n|n\right)}+{\tilde{\xi }}_{\left(n|n\right)}+w_{\left(n\right)} \end{array}$$

Error covariance of the Taylor expansion of REKF:

$$\begin{array}{c} P_{n+1|n}\left(REKF\right)=E\left\{{\tilde{x}}_{n+1|n}{{\tilde{x}}^{T}}_{n+1|n}\right\}\\ \;\;\;\;\;\;\;\;\;\;\;\;\;\;\;\;\;\;\;\;\;=A_{n+1|n}P_{n|n}A_{n+1|n}^{T}+P_{n|n}\left(\xi \xi \right)+{\hat{\xi }}_{n|n}{{\hat{\xi }}^{T}}_{n|n}+Q_{n} \end{array}$$

From this, we attain:

$$\Delta P_{n+1|n}=P_{n+1|n}\left(EKF\right)-P_{n+1|n}\left(REKF\right)={\hat{\xi }}_{n|n}{{\hat{\xi }}^{T}}_{n|n}$$

Since ${\hat{\xi }}_{n|n}{\hat{\xi }}_{n|n}^{T}$ is greater than or equal to 0, and ${\hat{\xi }}_{n|n}{\hat{\xi }}_{n|n}^{T}$ only when the matrix is orthogonal to ${\hat{\xi }}_{n|n}{\hat{\xi }}_{n|n}^{T}$.

$$\Delta P_{n+1|n}\geq 0$$

Thus, the error covariance matrix of REKF is minimized.

Through comparison, it can be observed that the ECM of REKF is minimized, achieving the highest accuracy. REKF extracts more useful information during the prediction phase, resulting in a reduction in the prediction error covariance matrix. Compared to the conventional EKF, REKF shows higher accuracy in the prediction phase.

## 4. The Error of REKF Converges to the True Value for Different Initial States

The state prediction error:

$${\tilde{x}}_{n+1|n}=A_{n+1|n}{\tilde{x}}_{n|n}+{\tilde{\xi }}_{n}+w_{n}$$

Then, there is the error covariance matrix:

$$\begin{array}{c} P_{n+1|n}=E\left\{{\tilde{x}}_{n+1|n}{\tilde{x}}_{n+1|n}^{T}\right\}\\ \;\;\;\;\;\;\;\;\;\;=A_{n+1|n}P_{n|n}{A^{T}}_{n+1|n}+E\left[{\tilde{\xi }}_{n}{\tilde{\xi }}_{n}^{T}\right]+Q+E\left[\left(A_{n+1|n}{\tilde{x}}_{n|n}\right){\left({\tilde{\xi }}_{n}\right)}^{T}\right] \end{array}$$

Since the cross-term simultaneously contains the state error ${\tilde{x}}_{n|n}$ and the system linearization error ${\tilde{\xi }}_{n}$, the Young’s inequality is utilized for decoupling.

Expand or contract the cross term $E[\left(A_{n+1|n}{\tilde{x}}_{n|n}\right){\left({\tilde{\xi }}_{n}\right)}^{T}]$

$$\begin{array}{c} E\left[\left(A_{n+1|n}{\tilde{x}}_{n|n}\right){\left({\tilde{\xi }}_{n}\right)}^{T}\right]\leq \alpha \cdot E\left[\left(A_{n+1|n}{\tilde{x}}_{n|n}\right){\left(A_{n+1|n}{\tilde{x}}_{n|n}\right)}^{T}\right]+\frac{1}{\alpha }E\left[\left({\tilde{\xi }}_{n}\right){\left({\tilde{\xi }}_{n}\right)}^{T}\right]\\ \;\;\;\;\;\;\;\;\;\;\;\;\;\;\;\;\;\;\;\;\;\;\;\;\;\;\;\;\;\;\;\;\;\;=\alpha A_{n+1|n}P_{n|n}A_{n+1|n}^{T}+\frac{1}{\alpha }P_{\xi \xi } \end{array}$$

Substitute it into the expression of error covariance:

$$\begin{array}{c} P_{n+1|n}\leq A_{n+1|n}P_{n|n}{A^{T}}_{n+1|n}+P_{\xi \xi }+Q+\alpha A_{n+1|n}P_{n|n}A_{n+1|n}^{T}+\frac{1}{\alpha }P_{\xi \xi }\\ =\left(1+\alpha \right)A_{n+1|n}P_{n|n}{A^{T}}_{n+1|n}+\left(1+\frac{1}{\alpha }\right)P_{\xi \xi }+Q \end{array}$$

By adjusting α, it can be ensured that the constant ρ<1 and the positive definite matrix ∏ satisfy:

$$\left(1+\alpha \right)A\prod A^{T}-\rho \prod +\left(1+\frac{1}{\alpha }\right)\left(\eta^{2}\prod +Q\right)≺0$$

This inequality proves that the error covariance $P_{n|n}$ decays proportionally ρ at each step and eventually converges to a bounded set. The influence of the initial error is suppressed and will not be continuously magnified.

## 5. Simulation Experiment

The data are mainly analyzed using the RMSE (Root Mean Square Error), which is a commonly employed metric for measuring the variation of predicted values with true ones. It is obtained by calculating the mean of the squared prediction errors and then taking the square root. A smaller RMSE indicates better model performance, as it represents the average difference between the predicted values and the true values. Specifically, the RMSE is the square root of the mean squared error, and it can be used to evaluate the accuracy of regression models.

$$RMSE=\sqrt{\frac{1}{n}\sum_{i=1}^{n}{\left(y_{i}-\hat{y_{i}}\right)}^{2}}$$

### 5.1. Simulation Example

To verify that REKF improves accuracy over the traditional EKF, a simulation experiment is conducted in MATLAB, given a class of nonlinear systems:

Simulation Experiment: Provide a state equation that can adjust the degree of nonlinearity, with the measurement equation being an under-measured linear equation.

For each considered case, 100 independent Monte Carlo runs were performed in MATLAB. Each run covered 100 time steps under the same parameter setting with independently generated noise realizations. The trajectory plots and cumulative RMSE curves reported in this section were obtained by averaging the corresponding results at each time step over the 100 runs.

$$x_{n+1}=\left[\begin{array}{c} x_{n+1}^{\left(1\right)}\\ x_{n+1}^{\left(2\right)} \end{array}\right]=\left[\begin{array}{c} 0.8\sin\left(\varphi x_{n}^{\left(1\right)}\right)+0.8\cos\left(\gamma x_{n}^{\left(2\right)}\right)\\ \sin\left(x_{n}^{\left(1\right)}\right)+\cos\left(x_{n}^{\left(2\right)}\right) \end{array}\right]+\left[\begin{array}{c} \omega_{n}^{\left(1\right)}\\ \omega_{n}^{\left(2\right)} \end{array}\right]$$

$$y_{n+1}=\left[\begin{array}{c} y_{n+1}^{\left(1\right)}\\ y_{n+1}^{\left(2\right)} \end{array}\right]=\left[\begin{array}{c} {x^{2}}_{n+1}^{\left(1\right)}\\ {x^{2}}_{n+1}^{\left(2\right)} \end{array}\right]+\left[\begin{array}{c} \upsilon_{n+1}^{\left(1\right)}\\ \upsilon_{n+1}^{\left(2\right)} \end{array}\right]$$

where φ and γ are used to adjust the degree of nonlinearity in the state equation.

Where $x_{n+1}={\left[x_{n+1}^{\left(1\right)},x_{n+1}^{\left(2\right)}\right]}^{T}$, $y_{n+1}={\left[y_{n+1}^{\left(1\right)},y_{n+1}^{\left(2\right)}\right]}^{T}$, $\omega_{n}$, as well as $\upsilon_{n+1}$ are independent white noises obeying a normal distribution in the system, with the original model inputs being the initial values of Q = diag{0.1,0.1} and R = diag{0.01,0.01}. Assume that the initial state input of the original model is ${\hat{x}}_{0}={\left[0.7,0.7\right]}^{T}$, and the initial estimated error covariance matrix is $P_{0}=I\in R^{2x2}$. Using the Extended Kalman Filter (EKF), the Unscented Kalman Filter (UKF), the Extended Kalman Filter with Remainder Terms without correlation terms (REKF-NC), and the Extended Kalman Filter with Remainder Terms (REKF), a comparison is made to estimate the target states $x_{1}$ and $x_{2}$.

#### 5.1.1. Weak Nonlinearity State Equation

The nonlinearity of the state equation is controlled by the parameters φ and γ. In the case of φ=γ=0.2, the system operates in a weakly nonlinear regime. The corresponding results are used to examine the estimation performance of different filtering methods under weak nonlinearity.

**Figure 2:**
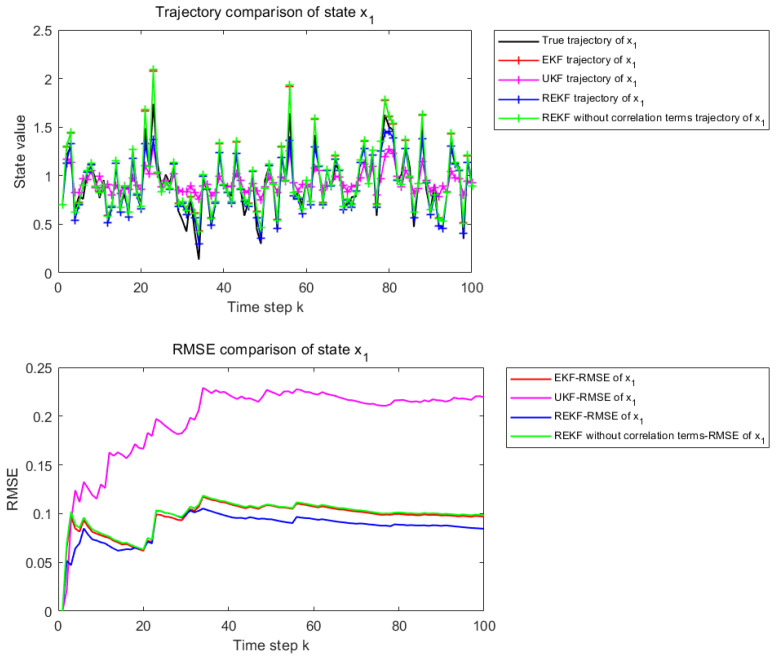
State estimation results for $x_{1}$ when φ and γ are set to 0.2.

**Figure 3:**
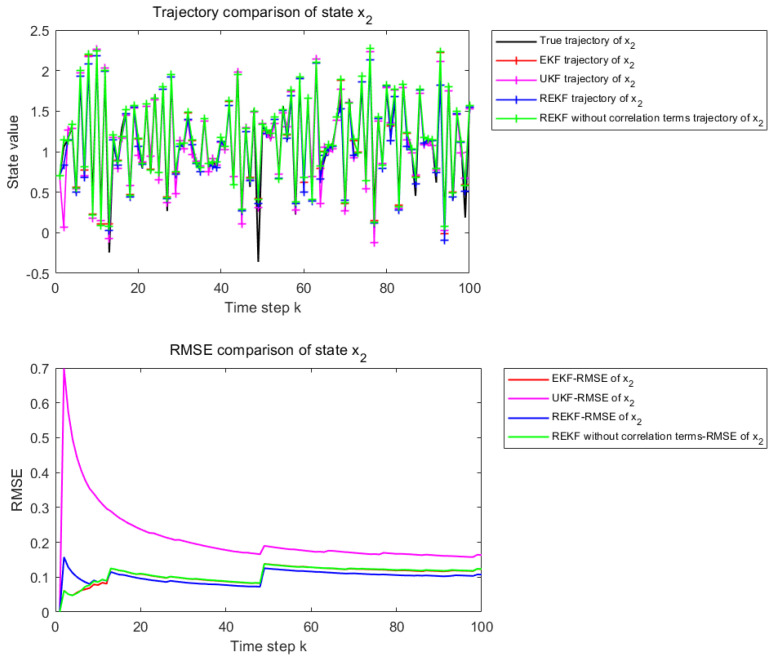
State estimation results for $x_{2}$ when φ and γ are set to 0.2.

Figure 2 and Figure 3 present the state estimation results over 100 time steps under the considered nonlinear setting. Figure 2 corresponds to the estimation performance for state $x_{1}$, whereas Figure 3 corresponds to that for state $x_{2}$. Each figure consists of two subplots. The upper subplot shows the trajectory comparison between the true state and the estimates obtained by EKF, UKF, the proposed REKF, and REKF without correlation terms, which provides a qualitative evaluation of the tracking capability of each method over the entire estimation horizon. The lower subplot presents the cumulative Root Mean Square Error (RMSE) curves of the corresponding methods over the same 100 time steps, thereby providing a quantitative comparison of the estimation accuracy and error accumulation behavior.

Under the weakly nonlinear setting, all methods are able to follow the overall variation trend of the states, while slight differences can still be observed in local tracking accuracy. The proposed REKF remains closer to the true state trajectory and generally yields the lowest cumulative RMSE among the compared methods. However, the performance gain in this case is relatively limited, since conventional filters are still effective when the system nonlinearity is weak.

Table 1 shows the RMSE values of EKF, UKF, REKF-NC, and REKF under the weakly nonlinear setting, together with the improvement rates of REKF relative to the other compared methods. For state $x_{1}$, the proposed REKF achieves an RMSE of 0.0594, corresponding to improvements of 13.07%, 66.10%, and 14.62% over EKF, UKF, and REKF-NC, respectively. For state $x_{2}$, the RMSE of REKF is 0.0599, which corresponds to improvements of 18.95%, 36.89%, and 20.69% over EKF, UKF, and REKF-NC, respectively. These results indicate that, even under weak nonlinearity, the proposed REKF still provides the best overall estimation accuracy among the compared methods.

#### 5.1.2. Moderate Nonlinearity State Equation

The nonlinearity of the state equation is controlled by the parameters φ and γ. In the case of φ=γ=0.4, the system operates in a moderately nonlinear regime. The corresponding results are used to examine the estimation performance of different filtering methods under moderate nonlinearity.

**Figure 4:**
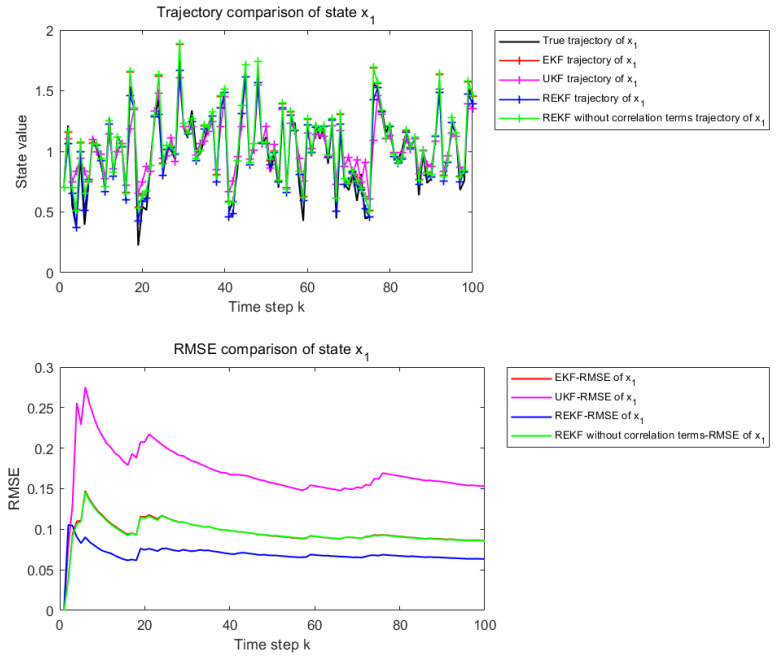
State estimation results for $x_{1}$ when φ and γ are set to 0.4.

**Figure 5:**
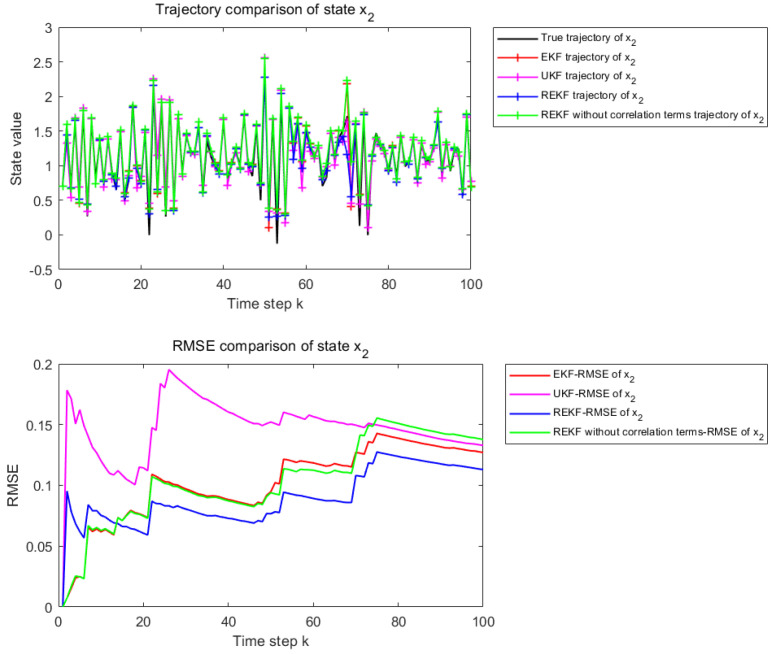
State estimation results for $x_{2}$ when φ and γ are set to 0.4.

Figure 4 and Figure 5 present the state estimation results over 100 time steps under the considered nonlinear setting. Figure 4 corresponds to the estimation performance for state $x_{1}$, whereas Figure 5 corresponds to that for state $x_{2}$. The subplot description is the same as in Figure 2 and Figure 3.

Under the moderately nonlinear setting, the differences among the compared methods become more evident. Although EKF, UKF, and REKF without correlation terms can still track the general state evolution, their local estimation errors increase noticeably. In contrast, the proposed REKF shows better trajectory consistency and maintains a lower cumulative RMSE, indicating that its advantage becomes more apparent as the nonlinearity increases.

Table 2 shows the RMSE values of EKF, UKF, REKF-NC, and REKF under the moderately nonlinear setting, together with the improvement rates of REKF relative to the other compared methods. For state $x_{1}$, the proposed REKF achieves an RMSE of 0.0487, corresponding to improvements of 22.14%, 53.60%, and 22.51% over EKF, UKF, and REKF-NC, respectively. For state $x_{2}$, the RMSE of REKF is 0.0617, which corresponds to improvements of 19.97%, 26.95%, and 23.77% over EKF, UKF, and REKF-NC, respectively. Compared with the weakly nonlinear case, the performance advantage of the proposed REKF becomes more evident under moderate nonlinearity.

#### 5.1.3. Strong Nonlinearity State Equation

The nonlinearity of the state equation is controlled by the parameters φ and γ. In the case of φ=γ=1, the system operates in a strongly nonlinear regime. The corresponding results are used to examine the estimation performance of different filtering methods under strong nonlinearity.

**Figure 6:**
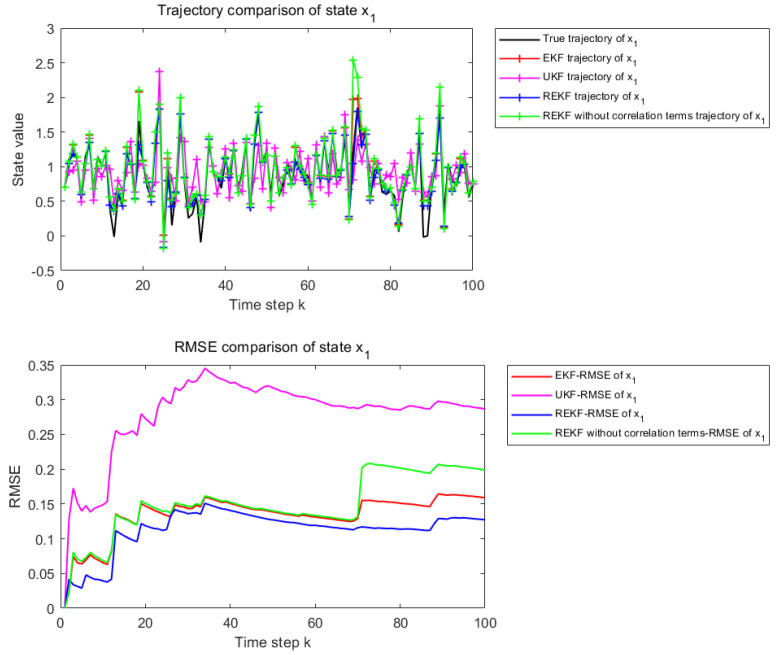
State estimation results for $x_{1}$ when φ and γ are set to 1.

**Figure 7:**
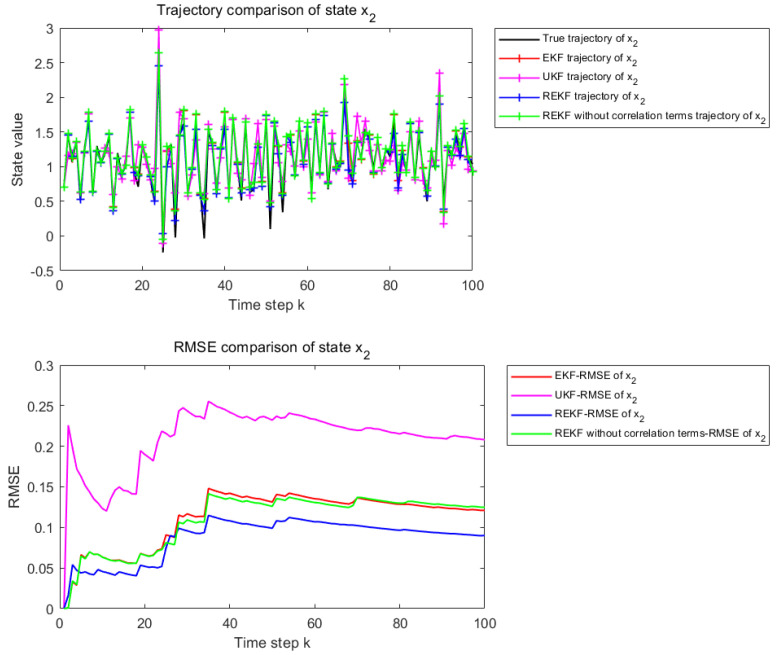
State estimation results for $x_{2}$ when φ and γ are set to 1.

Figure 6 and Figure 7 present the state estimation results over 100 time steps under the considered nonlinear setting. Figure 6 corresponds to the estimation performance for state $x_{1}$, whereas Figure 7 corresponds to that for state $x_{2}$. The subplot description is the same as in Figure 2 and Figure 3.

Under the strongly nonlinear setting, the superiority of the proposed REKF becomes much more pronounced. The compared methods exhibit larger deviations from the true state trajectory, and their cumulative RMSE increases significantly. By contrast, REKF still preserves better estimation stability and accuracy, demonstrating that remainder-term compensation and correlation modeling are particularly effective when the system is subject to strong nonlinear effects.

Table 3 reports the RMSE values of EKF, UKF, REKF-NC, and REKF under the strongly nonlinear setting, together with the improvement rates of REKF relative to the other compared methods. For state $x_{1}$, the proposed REKF achieves an RMSE of 0.0801, corresponding to improvements of 20.23%, 65.06%, and 28.42% over EKF, UKF, and REKF-NC, respectively. For state $x_{2}$, the RMSE of REKF is 0.0557, which corresponds to improvements of 27.05%, 63.88%, and 30.07% over EKF, UKF, and REKF-NC, respectively. Compared with the moderately nonlinear case, the superiority of the proposed REKF becomes even more pronounced under strong nonlinearity.

## 6. Results

With the rise in the degree of nonlinearity in the state equation, the accuracy of the traditional Extended Kalman Filter (EKF) gradually decreases when handling nonlinear systems. This is because the conventional EKF approximates the nonlinearities using a first-order Taylor expansion, neglecting higher-order nonlinear terms, which prevents the model from accurately capturing the complex dynamics of the system, especially when the nonlinearity is high. The limitations of this linear approximation cause the traditional EKF to fall short in terms of accuracy and stability.

In contrast, the Extended Kalman Filter with Remainder Expansion (REKF) effectively addresses this issue. By introducing remainder terms to replace higher-order terms, this method can more accurately model the nonlinear behavior of the system. The remainder terms provide a higher-order approximation of the nonlinear components, thus reflecting the system’s dynamic changes more precisely and reducing errors caused by linear approximation. In highly nonlinear cases, this method significantly improves the accuracy of state estimation and enhances the stability of the model.

In addition to EKF, the Unscented Kalman Filter (UKF) is also included as a comparative method. In the present experiment, UKF performs worse than both EKF and the proposed REKF. A plausible reason is that the quadratic measurement function introduces an even mapping, under which the contributions of symmetric sigma-point pairs to the state-measurement cross-covariance may partially cancel, thereby weakening the Kalman gain. Moreover, the UKF parameter setting used here may not be well suited to the considered model. Therefore, the inferior performance of UKF in this case is likely model-dependent rather than a general conclusion. Under the considered conditions, the proposed REKF maintains a clearer advantage in both trajectory tracking accuracy and cumulative RMSE performance, and this advantage becomes more evident as the nonlinearity increases. In terms of computational complexity, EKF has the lowest cost among the compared methods. UKF is more computationally demanding because it requires the generation and propagation of sigma points. REKF introduces additional computations for remainder-term identification and correlation-aware covariance updates, so its complexity is higher than that of EKF, but it still preserves the recursive filtering structure.

Experimental results show that, as the degree of nonlinearity increases, the accuracy of the Extended Kalman Filter with remainder terms improves more significantly compared to traditional EKF. Moreover, the improvement of REKF over UKF and REKF without correlation terms also becomes more pronounced as the system nonlinearity increases. This indicates that the REKF method is better suited for handling complex nonlinear systems, particularly in long-duration runs or noisy environments, where it provides more stable and accurate state estimates. Furthermore, the use of remainder terms in the Kalman filter reduces the prediction instability caused by the accumulation of linearization errors in traditional EKF, making the model more robust in nonlinear systems.

Therefore, the Extended Kalman Filter with remainder expansion shows significant advantages in both accuracy and stability, compared with EKF, UKF, and REKF without correlation terms, particularly when dealing with highly nonlinear or noisy systems, providing more reliable state estimation. This result is of great significance for Nonlinear State Monitoring and Soft Sensing, where it can improve the performance and robustness of these systems.

## 7. Discussion

In this paper, we design an Extended Kalman Filter with Remainder Terms Considering Correlations (REKF). Experimental results demonstrate that REKF outperforms the traditional EKF in terms of estimation accuracy for nonlinear Gaussian systems. By incorporating higher-order remainder terms, the proposed method achieves improved accuracy compared with second-order approximations, while also exhibiting enhanced stability. In addition, the proposed approach accounts for truncation in the state equation and correlation terms in the measurement equation. The superiority of the algorithm is verified through a gradually enhanced nonlinear system, showing its effectiveness under increasing nonlinearity. These results indicate that the proposed REKF has significant potential in networked sensing environments where nonlinear effects cannot be neglected.

From a practical perspective, it is important to note that the advantage of the proposed REKF depends on the degree of system nonlinearity. When the system exhibits weak nonlinearity, the conventional EKF is generally sufficient to achieve satisfactory performance, and the improvement provided by REKF is limited. However, as the nonlinearity becomes stronger, the truncation errors introduced by the Taylor series approximation in EKF become increasingly significant. In such cases, the proposed REKF, which explicitly compensates for higher-order remainder terms and accounts for correlation effects, can provide more accurate and stable state estimation. Therefore, the use of REKF is particularly justified in scenarios where strong nonlinearity or model mismatch leads to non-negligible approximation errors.

It should also be noted that the simulation example considered in this paper is constructed as a controlled benchmark, rather than a direct representation of a specific engineering system. The primary objective is to isolate the impact of increasing nonlinearity and the associated truncation errors arising from Taylor series approximation, which are the key factors addressed by the proposed REKF. Although the model does not correspond to a particular real-world system, its structure reflects a class of problems involving nonlinear state estimation under limited measurement conditions. Therefore, the insights obtained from the simulation provide meaningful guidance for understanding the behavior of filtering algorithms in strongly nonlinear scenarios.

Future work will focus on validating the proposed method in practical applications, such as battery state-of-charge estimation, soft sensing in chemical processes, and nonlinear target tracking or navigation systems. In these scenarios, strong nonlinearity and model mismatch are common, making them suitable testbeds for further evaluating the effectiveness of the proposed REKF framework.

In addition, the proposed REKF can in principle be embedded into output-feedback or observer-based closed-loop control systems. Its benefit is expected in scenarios where controller performance is sensitive to estimation accuracy under strong nonlinearity. However, the analysis of closed-loop stability considering the interaction between the controller and the filter is beyond the scope of this paper and will be investigated in future work.

## Figures and Tables

**Table 1:** REKF improvement rate.

Filter	RMSE−*x*_1_	RMSE−*x*_2_	Improvement Rate of REKF−*x*_1_	Improvement Rate of REKF−*x*_2_
EKF	0.0684	0.0739	13.07%	18.95%
UKF	0.1753	0.0950	66.10%	36.89%
REKF-NC	0.0696	0.0756	14.62%	20.69%
REKF	0.0594	0.0599	–	–

**Table 2:** REKF improvement rate under moderate nonlinearity.

Filter	RMSE−*x*_1_	RMSE−*x*_2_	Improvement Rate of REKF−*x*_1_	Improvement Rate of REKF−*x*_2_
EKF	0.0625	0.0771	22.14%	19.97%
UKF	0.1049	0.0844	53.60%	26.95%
REKF-NC	0.0628	0.0809	22.51%	23.77%
REKF	0.0487	0.0617	–	–

**Table 3:** REKF improvement rate under strong nonlinearity.

Filter	RMSE−*x*_1_	RMSE−*x*_2_	Improvement Rate of REKF−*x*_1_	Improvement Rate of REKF−*x*_2_
EKF	0.1004	0.0764	20.23%	27.05%
UKF	0.2291	0.1543	65.06%	63.88%
REKF-NC	0.1118	0.0797	28.42%	30.07%
REKF	0.0801	0.0557	–	–

## Data Availability

Data are contained within the article.

## Abbreviations

The following abbreviations are used in this manuscript:

EKF	Extended Kalman Filter
REKF	Extended Kalman Filter with Remainder Terms Considering Correlations
RMSE	Root Mean Square Error
